# Characteristics of BCR–ABL gene variants in patients of chronic myeloid leukemia

**DOI:** 10.1515/med-2021-0309

**Published:** 2021-06-23

**Authors:** Huma Amin, Suhaib Ahmed

**Affiliations:** Department of Pathology, Rawalpindi Medical University, Rawalpindi, Pakistan; Department of Pathology, Islamic International Medical College, Rawalpindi, Pakistan

**Keywords:** BCR–ABL, chronic myelyoid leukemia, fusion transcript, major breakpoint cluster region, reverse transcriptase PCR

## Abstract

**Background:**

Depending on breakpoints of rearrangement different types of BCR–ABL fusion protein can be generated in patients of chronic myeloid leukemia (CML). The aim of this study is to observe frequencies of major transcripts in CML patients by reverse transcriptase polymerase chain reaction (RT-PCR) and their hematological features at the time of presentation.

**Materials and methods:**

This cross sectional study was performed at Molecular Lab of Riphah International University, Islamabad from January to June 2019. Consecutive peripheral blood samples of 70 newly diagnosed CML patients in chronic phase were analyzed by RT-PCR to detect different BCR–ABL transcripts. Routine blood cell counts were assessed by an automated hematology analyzer.

**Results:**

All samples expressed typical BCR–ABL rearrangement. Expression of either e14a2 or e13a2 transcript was detected in 38 (54%) and 30 (43%) patients, respectively. Coexpression of e13a2 + e14a2 was found in 2 (3%) patients. The mean total leukocyte count was higher in group expressing e13a2 (*P* = 0.01). Higher mean platelet count was noted in patients with e14a2 transcript, but this difference was statistically insignificant (*P* = 0.1). The association of male gender was observed with the group exhibiting e14a2 (*P* = 0.01). There was no statistically significant association between transcript type and different ranges of age, hemoglobin levels, and platelet and total leukocyte counts (*P* > 0.05).

**Conclusion:**

e14a2 transcript was most common transcript in CML patients. Patients exhibiting e13a2 subgroup presented with significantly higher mean white blood cell count at the time of presentation. Significantly higher proportion of male patients was found to express e14a2 transcript over e13a2.

## 1 Introduction

CML is associated with a cytogenetic abnormality known as Philadelphia (Ph) chromosome. This is one of the definitive diagnostic markers for CML [1]. Ph chromosome arises from a reciprocal translocation t (9; 22) between chromosome 9 and 22 [2,3]. The Philadelphia chromosome is present in approximately 95% of patients with CML [4]. However, few CML patients do not demonstrate Ph chromosome or might have normal karyotype, but even in one third of these patients, there is occult BCR–ABL fusion gene (Ph chromosome negative and BCR–ABL positive) [5]. Molecular analysis confirms presence of BCR–ABL in these patients [6].

**Figure 1:**
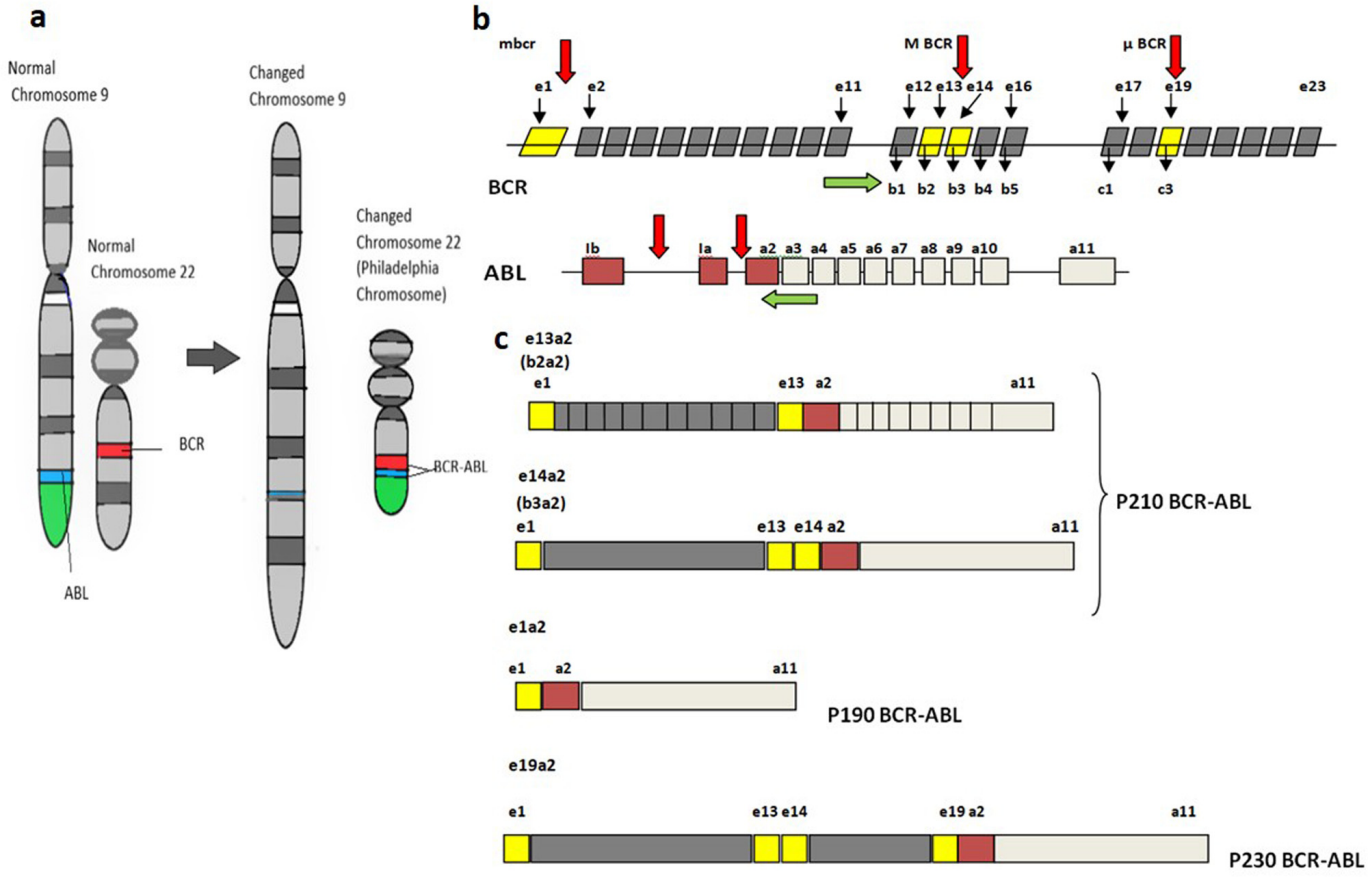
(a) Schematic diagram of the normal structure of chromosome 9 and 22 and translocation between chromosome 9 and 22, resulting in Philadelphia chromosome. (b) Schematic representation of the molecular structure of BCR and ABL genes. Red arrows indicate breakpoints in both genes. Green arrows show the placement of forward and reverse primer on the exons of BCR and ABL for detection of different transcripts by RT-PCR. (c) Schematic diagram of the structure of chimeric mRNA transcribed from different breakpoints of BCR–ABL fusion gene.

Break point in BCR gene occurs in three main regions: Major (M-BCR), minor (m-BCR), and micro (µ-BCR) breakpoint cluster regions [3]. Large majority of CML patients have breakpoints in M-BCR region on chromosome 22 [7]. This area consists of *BCR* exons 12–16 (previously referred to as exons b1–b5, respectively) [8] (Figure 1). The classic transcript found in majority of CML patients is b2a2 or b3a2, formed by fusing exon 13 (b2) or exon 14 (b3) of BCR to exon 2 (a2) of ABL gene, respectively. Both of them code for a 210 kDa (p210) novel protein [9]. More than 95% of *BCR*–*ABL* transcripts are either e13a2 or e14a2 in CML. “Atypical” transcripts with a breakpoint in *ABL* intron 1 and *BCR* intron 6 (e6a2) and breakpoint in *ABL* intron 2 and *BCR* intron 1 (e1a3), *BCR* intron 13 (e13a3), and *BCR* intron 14 (e14a3) have been sometimes reported [10]. Variable frequencies of these fusion transcripts in CML patients are observed around the world [11].

Clinical and hematological profile of CML patients with different fusion transcripts revealed some noticeable features [12]. The knowledge of differences in breakpoints might have some clinical importance, and disease phenotype of patients may vary with them. M-BCR usually presents with classical CML phenotype [11]. While an interesting finding associated with b3a2 fusion transcript is the higher platelet counts [13].

Reverse transcriptase PCR (RT-PCR) is one of the most sensitive techniques used for detection of BCR–ABL transcripts associated with CML [9]. Most of the current RT-PCR methods are designed and optimized for detecting the transcript of M-BCR and m-BCR breakpoints. Very large PCR products or atypical transcripts may get away with detection using these routine primers. The application of RT-PCR techniques can be improved by several methods including the development of multiplex RT-PCR to detect the BCR–ABL variants in patients with CML [14].

In this study, RT-PCR was used for the detection of all major *BCR*–*ABL* transcripts in CML patients, and the frequency of BCR–ABL transcripts was observed along with their presenting hematological features.

## 2 Material and methods

This cross-sectional study was carried out from January 2019 to June 2019. Patients were recruited from tertiary care hospital of Rawalpindi. Molecular analysis was carried out in the laboratory settings of Riphah International University. Informed consent was taken from each patient. The study was approved by the ethical review committee of the University.

Total of 70 consecutive newly diagnosed patients of CML in chronic phase irrespective of age and gender were included in this study. Diagnosis of CML was made based on clinical presentation and morphologic criteria of bone marrow aspirate. Patients who had started any kind of treatment previously with any dose or for any length of time were excluded from this study.

Peripheral blood samples in ethylene diamine tetra acetic acid (EDTA) tubes were collected and rapidly transported at 2–8°C to the laboratory to minimize mRNA degradation. Routine, blood cell counts were performed on automated hematology analyzer.

### 2.1 RNA extraction and cDNA synthesis

Extraction of total RNA was carried from whole blood by TRIzol^®^ reagent (TRI Reagent L.S). The extracted RNA was reverse-transcribed to cDNA by using gene-specific primer (ABL-2). cDNA was used as a template for qualitative detection of type of BCR–ABL transcript by one-step RT-PCR.

### 2.2 RT-PCR conditions

Primer sequences to be used for qualitative RT-PCR are given below. Primers were obtained from Integrated DNA Technologies (IDT) in the lyophilized form and stored at −20°C.

Forward primer: BCR E-12 AGA ACA TCC GGG AGC AGC AGA AGA A; Reverse primer: ABL-2 TCC AAC GAG CGG ATT CAC T.

DNA amplification was done in 30 μL reaction mixture, which included 15 μL PCR Mix (Invitrogen PCR Super Mix includes PCR buffer (22 mM Tris HCL, pH 8.4), 1.65 mM MgCL_2_, 220 µM dNTPs, and 22 U recombinant *Taq*DNA polymerase/mL), 1 μL of 10 pM Primer Mix (forward and reverse primers), 0.25 μL enzyme RT (SuperScript™ III Reverse Transcriptase 200 U/μL Invitrogen, USA), 5 μL template RNA, and 9 μL nuclease-free water. The synthesis of complementary DNA and amplification of the target were done by using the recommended thermal cycling conditions outlined below:

\begin{array}{c}\text{Reverse}\hspace{.5em}\text{transcription}\hspace{.5em}\text{at}\hspace{.5em}\text{50}^\circ \text{C}\hspace{.5em}\text{for}\hspace{.5em}\text{40}\hspace{.25em}\text{min}\hspace{.5em}\text{for}\hspace{.5em}\text{one}\hspace{.5em}\text{cycle}\\ \left.\begin{array}{c}\text{Denaturation}\hspace{.5em}\text{at}\hspace{.5em}\text{94}^\circ \text{C}\hspace{.5em}\text{for}\hspace{.5em}\text{15}\hspace{.25em}\text{s}\\ \text{Annealing}\hspace{.5em}\text{at}\hspace{.5em}\text{63}^\circ \text{C}\hspace{.5em}\text{for}\hspace{.5em}\text{30}\hspace{.25em}\text{s}\\ \text{Extension}\hspace{.5em}\text{at}\hspace{.5em}\text{72}^\circ \text{C}\hspace{.5em}\text{for}\hspace{.5em}\text{45}\hspace{.25em}\text{s}\end{array}\right\}\text{30}\hspace{.5em}\text{cycles}\end{array}]

Amplified products were electrophoresed on a 6% polyacrylamide gel electrophoresis. The gels were stained with 0.1% silver nitrate. The bands were detected at 310 and 385 bp for e13a2 and e14a2, respectively. Molecular variants of e1a2 and e19a2 products were expected to form bands at 481 and at 244 bp, respectively.

To determine the sensitivity of the PCR method, an assay was carried out by generating a 10-fold (1/1, 1/10, 1/100, and 1/1,000) of a RT-PCR positive BCR–ABL RNA in nuclease-free water. These serial dilutions and one negative sample were run under identical RT-PCR conditions (as described earlier) in five separate reaction tubes for cDNA synthesis and PCR amplifications.

### 2.3 PCR strategy

Initial PCR was performed with E12 forward and ABL 2 reverse primers to detect p210 kDa variants (Figure 1). Since atypical transcripts are rare, a second confirmatory PCR was to be performed in typical variant *BCR*–*ABL*-negative patient. Atypical transcripts could be easily suspected from their different product sizes on gel electrophoresis after preliminary PCR results. Second PCR was planned to be performed using the forward primers (E1, E6, E19, etc.) with common reverse ABL3 primers separately for the suspected breakpoint region. To further validate the finding, atypical transcript could be confirmed by DNA sequencing.

All statistical calculations were done using SPSS 22 software. Quantitative data were expressed as mean ± S.D. The variables included numerical data such as age, Hb concentration, WBC count, and platelet count. Independent *t* test was applied to compare the means of two different transcripts. Frequency and percentages were calculated for the qualitative data such as gender and type of transcript. Variables such as age, hemoglobin, WBC counts, and platelet counts were also presented as ranges, and their frequencies were calculated. Chi square test was performed to see the association of categorical variables with different transcripts. *P* value of <0.05 was considered as significant.


**Ethics approval and consent to participate:** The study was approved by the ethical review committee of Institutional review committee Islamic International Medical College (Ref No. Appl. # Riphah/ERC/15/0119).
**Patient consent for publication:** Consent was obtained from all the patients for the use of their medical data for research purposes.

## 3 Results

Molecular analysis was performed with conventional RT-PCR on total of 70 patients (64% males and 36% females) for the type of CML transcript. Demographic details of these patients are presented in Table 1.

**Table 1:** Clinical and laboratory details of CML patients in study

Number of patients (*n*)	70
Mean age (years)	41.10 ± 14.14
Range of age (years)	15–70
Male/female (*n*)	45/25
Male-to-female ratio	1.8
Mean leukocytes count (×10^9^/L)	145.12 ± 89.03
Mean hemoglobin levels (g/dL)	10.65 ± 2.42
Mean platelets count (×10^9^/L)	441.77 ± 403.18

CML: chronic myeloid leukemia.

Clinical and laboratory data of patients in chronic phase at the time of presentation. Data are presented as mean ± standard deviation.

### 3.1 Qualitative analysis of BCR–ABL transcripts

RT-PCR was carried out with the aim that it should be able to primarily detect the most prevalent transcripts e13a2 and e14a2. An internal control (ABL gene) reaction was also included to correctly interpret negative results.

Initial PCR for the M-BCR transcripts was performed with BCR forward primer located on exon 12 (e12). The common reverse ABL primer was located in ABL exon 2 (Figure 1). This primer combination in RT-PCR can simultaneously detect both common types of BCR–ABL breakpoint (e14a2 and e13a2) on the M-BCR region in single reaction.

**Figure 2:**
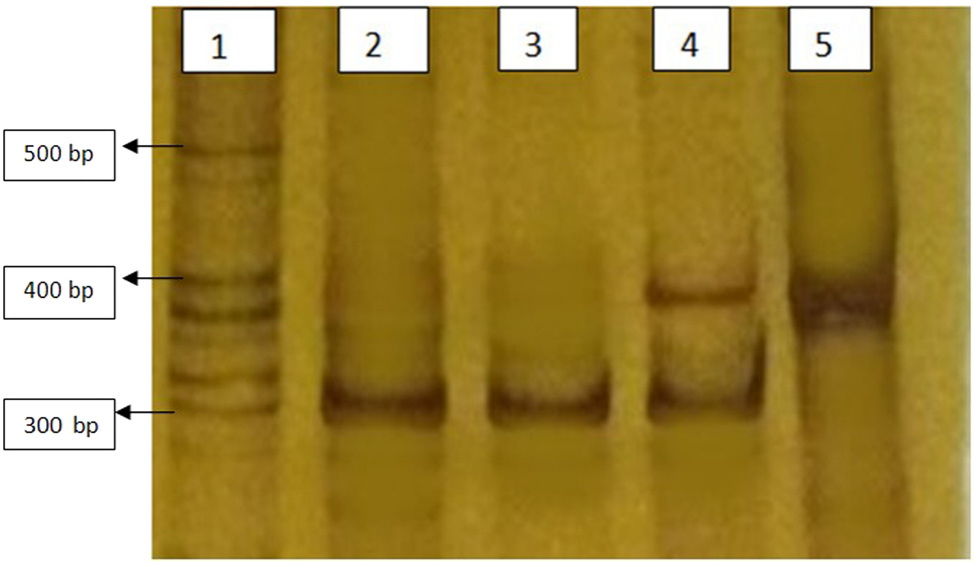
Silver stained polyacrylamide gel electrophoresis showing different transcripts of BCR–ABL after RT-PCR with e12a2 primer. All lanes are positive for BCR–ABL mutation typical p210 kDa (e13a2 and/or e14a2) transcripts. Lane 1 shows ladder (molecular weight marker) of various size fragments. bp (base pair). Lanes 2 and 3 are from chronic phase CML patients expressing 310 bp fragment of e13a2 BCR–ABL. Lane 4 is from a chronic phase CML patient co expressing both e13a2 and e14a2 BCR–ABL. Lane 5 is from a chronic phase CML patient expressing 385 bp fragment of e14a2 BCR–ABL.

All studied patients 70/70 (100%) were found positive for typical BCR–ABL (p210 kDa). Either e13a2 or e14a2 transcript was detected in all the samples. The detected bands were as follows: 385 bp for e14a2 and 310 bp for e13a2. No atypical transcript was observed in any of the sample analyzed. Neither product of unexpected size was detected on gel electrophoresis after conventional RT-PCR. Common forward primer e12 was only used in this study to reveal the type of transcripts. Figure 2 represents the results of RT-PCR for some of the patients.

Running the tenfold dilution on electrophoresis gel after RT-PCR revealed that the detection limit of this assay was up to 10^−4^ for each sample.

### 3.2 Frequency of different transcripts along with their presenting features in CML patients

**Figure 3:**
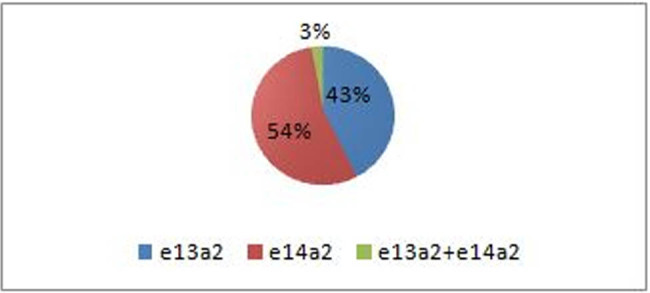
Frequency of different types of BCR–ABL transcripts among 70 CML patients in chronic phase analyzed in the study. Total of 38 (54%) patients had solitary expression of e14a2 transcript. Expression of e13a2 exclusively was found in 30 (43%) patients. Co-expression of both types of transcripts (e13a2 + e14a2) was observed in only two (3%) patients. The types of transcripts detected in all patients are color coded as shown in box.

Among 70 CML patients analyzed, e14a2 transcript was most commonly detected. Total of 38 (54%) cases had solitary expression of e14a2, and 30 (43%) of the remaining cases were positive for e13a2 transcripts exclusively. Co-expression of both types of transcripts (e13a2 + e14a2) was observed in only two (3%) patients and were excluded from further analysis (Figure 3).

The comparative analysis of quantitative data (expressed as means ± S.D) of the two groups with expression of either e13a2 or e14a2 transcript is presented in Table 2. Patients with e14a2 transcripts showed lower mean age, but the difference was not statistically significant (*P* = 0.4). The mean total white blood cell count was significantly higher in the group of patients expressing e13a2 than those with e14a2 transcript (*P* = 0.01). Higher mean platelet count was observed in patients with e14a2 transcript, but this difference was not statistically significant (*P* = 0.1). No significant difference in mean hemoglobin concentration between the two groups was found (*P* > 0.05; Table 2).

**Table 2:** Characteristics of the CML patients with e13a2 and e14a2 transcripts

Variables	e13a2	e14a2	*P* value
Mean age (years)	42.90 ± 13.82	40.13 ± 14.93	0.4
Mean leukocytes count (×10^9^/L)	173.75 ± 97.17	120.77 ± 77.67	0.01*
Mean hemoglobin levels (g/dL)	10.84 ± 2.62	10.50 ± 2.34	0.5
Mean platelets count (×10^9^/L)	352.33 ± 229.8	512.13 ± 495.78	0.1

CML: chronic myeloid leukemia.

Data on 68 CML patients in chronic phase at the time of presentation expressing either of the two transcripts. Co expression of transcripts was detected in only two patients (data not summarized). Data are presented as mean ± standard deviation. *P* < 0.05 was considered as significant.

*Statistically significant.

**Figure 4:**
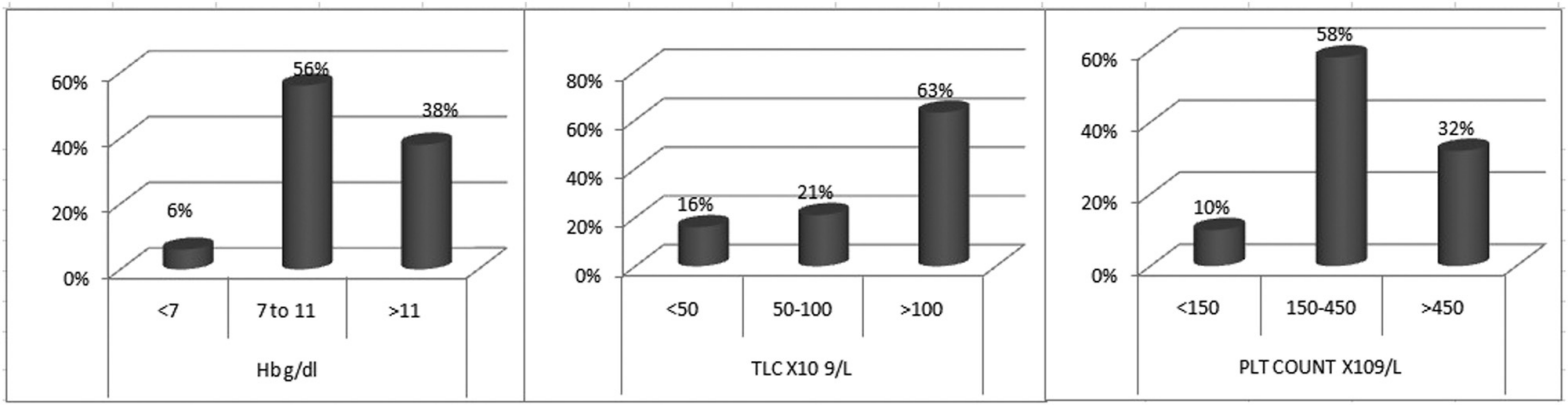
Frequency of CML patients with either e13a2 or e14a2 transcript in different hemoglobin levels, TLC (total leukocyte count), and PLT (platelets) count. Data on 68 patients in choric phase at the time of presentation were summarized.

Figure 4 represents the frequency of all study patients with typical p210 kDa (e13a2 or e14a2) transcripts in different ranges of hemoglobin concentration, leukocyte count, and platelet count. The highest frequency of 38 (56%) patients at the time of diagnosis had hemoglobin concentration in the range of 7–11 g/dL. The majority of patients of typical transcript (43 (63%) patients) presented with TLC ranging from more than 100 × 10^9^/L. The analysis of patients with typical transcript with different ranges of platelet count revealed that 39 (58%) patients had normal platelet count at the time of diagnosis followed by thrombocytosis in 22 (32%) patients (Figure 4).

Variables such as age, hemoglobin levels, and platelet and total leukocyte counts were further divided into ranges to see the distribution of patients with either e13a2 or e14a2 transcript. The comparison of categorical data in two transcripts group is summarized in Table 3. A significantly higher frequency of males 29 (76%) was observed in patients exhibiting e14a2 transcript compared to females (*P* = 0.01). Distribution of the transcript within two age groups showed no significant difference (*P* = 0.51). Similarly, no statistically significant difference in frequency of patients was observed in different ranges of hemoglobin levels and platelet and total leukocyte counts with specific transcript type (*P* > 0.05).

**Table 3:** Distribution of patients exhibiting e13a2 and e14a2 transcripts according to gender and different ranges of age, hemoglobin levels, and total leukocyte and platelets counts

Hematological variables	Transcript type		Total	*P* value
	e13a2 (*n* = 30)	e14a2 (*n* = 38)		
**Age (years)**				0.51
<45	15 (50%)	22 (58%)	37 (54%)	
45 and above	15 (50%)	16 (42%)	31 (46%)	
**Gender**				0.01*
Male	14 (47%)	29 (76%)	43 (63%)	
Female	16 (53%)	09 (24%)	25 (37%)	
**Hemoglobin levels (g/dL)**				0.61
<7 g/dL	1 (3%)	3 (8%)	4 (6%)	
7–11 g/dL	16 (53.3%)	22 (58%)	38 (56%)	
>11 g/dL	13 (43.3%)	13 (34%)	26 (38%)	
**Total leukocyte count 10** ^**9**^ **/L**				0.15
<50	2 (7%)	9 (24%)	11 (16%)	
50–100	6 (20%)	8 (21%)	14 (21%)	
>100	22 (73%)	21 (55%)	43 (63%)	
**Platelet count ×10** ^**9**^ **/L**				0.09
<150	5 (16%)	2 (5%)	7 (10%)	
150–450	19 (64%)	20 (53%)	39 (58%)	
>450	6 (20%)	16 (42%)	22 (32%)	

Chi square test was performed to see the association of categorical variables with different transcripts. *P* < 0.05 is taken as significant. Data are summarized from 68 patient with either e13a2 or e14a2 transcript.

*Statistically significant.

**Figure 5:**
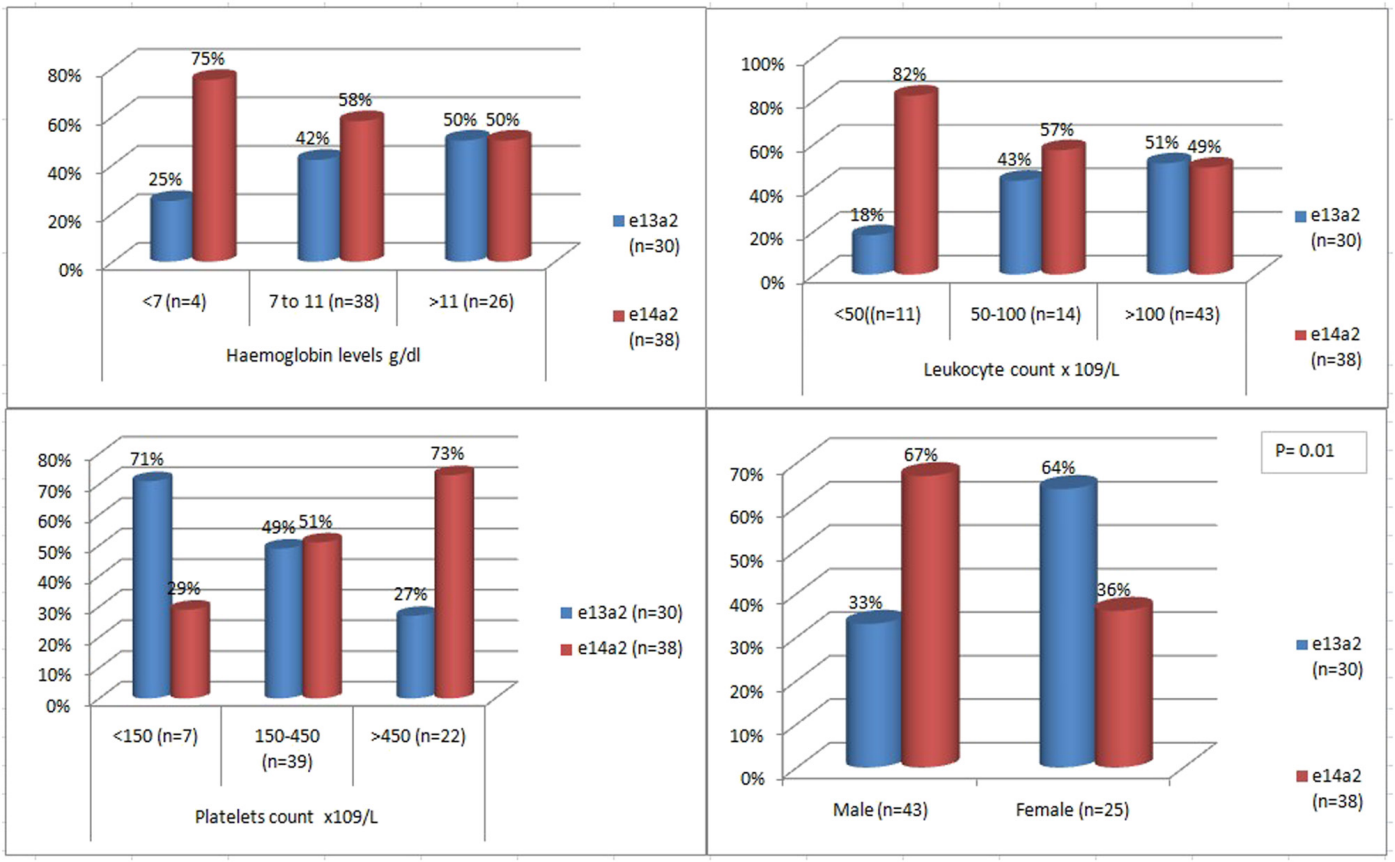
Transcript stratified frequency of CML patients within different subsets of hematological parameter and gender. Bar chart demonstrates association of these variables with e13a2 and e14a2 transcripts. The types of transcripts are color coded as shown in box. *P* value of <0.05 is taken as significant.

Figure 5 exhibits the relation of e13a2 and e14a2 transcripts within different subsets of hematological parameters and gender. Association of male gender with e14a2 and female gender with e13a2 can be appreciated (*P* = 0.01). Larger proportion of e14a2 transcript was demonstrated in subgroups of (i) platelets count of >450 × 10^9^/L and (ii) leukocyte count of <50 × 10^9^/L, but statistically significant association between these subgroups and transcript could not be established (*P* > 0.05).

## 4 Discussion

The aim of this study is to detect and analyze the demographic and hematological characteristics according to the variants of BCR–ABL transcript in CML patients in our local population.

The technique used for detection of various transcripts consists of performing RT-PCR by choosing a primer specific for exon b1 (e12) on the BCR gene and reverse primer on *ABL* exon 2. The present approach has the benefit of simplifying and shortening the procedure of RT‑PCR for detection of these transcripts in single reaction. The need of independent sequence-specific primers were eliminated, which increases the complexity, workload, and cost of PCR.

The mean age of CML patients at diagnosis in this study group was found to be 41.10 ± 14.14 years (range, 15–70 years). This is much younger when compared with data from the western literature, showing older mean age (66, 52, and 59 years) at diagnosis in CML patients [15,16,17]. However, this finding is consistent with other Asian studies reported earlier [18,19,20]. Young age at diagnosis in CML patients is usually associated with low- and middle-income countries, and some possible environmental factors and under reporting of geriatric population in these countries could be the possible explanation for this [21].

This study revealed higher prevalence of males (63%) compare to females (37%) in CML patients. The male-to-female ratio was observed to be 1.8:1. This is almost similar to already reported figures from local [20,22] as well as studies from around the world [16,17,23]. Male predominance could be possibly because of the fact that hematological neoplasms are usually more common in males compared to females owing to genetic and hormonal differences [21].

This study demonstrated that the predominant transcript was e14a2 (b3a2) in CML patients. Comparable frequencies of e13a2, e14a2, and their co-expression were documented in some studies, but divergence from these results was also observed. The frequencies of common transcripts in CML patients in different populations have been summarized in Table 4.

**Table 4:** Prevalence of BCR–ABL transcripts in different studies from various countries

Countries	e14a2(b3a2)	e13a2(b3a2)	e14a2 + e13a2	Others
Present study	54%	43%	3%	—
Bulgaria [13]	54%	45%	0%	—
India [24]	56.25%	41.25%	0%	2.5%
Pakistan [25]	66.7%	20.8%	0%	12.5%*
Brazil [26]	64%	34%	2%	—
Malaysia [27]	69%	31%	0%	—
Korea [28]	68%	32%	0%	—
Iran [29]	63%	20%	3%	14%
India [30]	68%	24%	0%	—
Argentina [31]	37.5%	41.7%	8.3%	12.5%
Syria [32]	14.3%	57%	—	28.7%
Pakistan [33]	26%	56%	—	18%
Brazil [34]	48%	36%	16%	—

Others include e1a2, e19a2, b3a3/b2a2, b3a3/b2a3, b3a3, and e1a3.

*No transcript detected.

A study conducted in India and Bulgaria calculated the ratio of the two major transcripts of M-BCR region e14a2:e13a2 to be 1.3:1, which is almost in agreement to the ratio observed in the present study [13,24]. However, studies from our local population represent comparatively higher ratio of the two transcripts than reported here [9,25]. Two times higher (ratio 2:1) of e14a2 than e13a2 were also reported in CML patients in Brazil, Malaysia, and Korea [26,27,28], while studies from Iran and India demonstrated the frequency of e14a2 transcripts to be almost three times higher than that of e13a2 [29,30]. Few studies have also documented higher frequency of e13a2 compared to more common e14a2 transcript in CML patients [31,32,33]. The variation in frequencies of common transcript among different population and regions of the world can be explained based on the dissimilarities of natural genetic factors, environmental factors, and living style in different ethnic groups [34].

This study demonstrated co expression of e13a2/e14a2 in only two patients (3%) of 70 patients analyzed. Varying results from all over the world have been reported regarding the co-expression of both transcripts e13a2 and e14a2 in patients with CML. Incidence of this co expression has been reported to be as high as 16% in a study from Brazil [34]. However, in our local population, the frequency of co-expression was reported to be very low, which is in agreement to the result of the present study [25,33].

The highest percentage (63%) of CML patient at the time of presentation in the present study had the total leukocyte count of >100 × 10^9^/L, which means greater tumor burden at the time of diagnosis. The majority (58%) of the study patients also had platelet count within normal range. Both these findings are in accordance to the previous studies [22,23].

Clinical features of the patients with two groups of typical transcript were not found to be starkly different in this study. When comparing the difference in means of numerical variable, it was found that patients with e14a2 transcript were younger than patients with e13a2 transcript although this difference was not statistically significant. A recent study from our local population [25] and report originating from the Syrian population endorsed this observation [35]. The only hematological variable significantly different in our study was higher mean WBC counts in the group of patients expressing e13a2 than in those with e14a2 transcript. Higher mean WBC count in patients carrying e13a2 transcript was also recognized in previous studies [26,36]. Many studies proved statistically significant association between e14a2 transcripts and higher mean platelets count in CML patients at the time of diagnosis [13,26,36,37]. Higher platelets count in patients with e14a2 transcript was also observed in this study, but the difference was not found to be statistically significant. Few other studies reported no differences in hematological parameters in both transcripts of BCR–ABL fusion gene [25, 34, 35].

While observing possible relationship between the BCR–ABL variants and clinical parameters in CML patient, significant association of male gender was observed in e14a2 transcript in this study. This finding is in accordance to the studies conducted in Iran, India, and Iraq [29,30,36]. However, other studies do not support this observation and report no significant association in male and female proportion in both types of fusion gene [13,28,35].

The association of fusion transcripts was also compared by dividing hemoglobin levels, TLC, and platelet count in different ranges. Although e14a2 did show a higher association with high platelet count compared to e13a2, but it was not found to be statistically significant (*P* = 0.09). Similarly by stratifying the patients on the basis of white blood cell (WBC) count and hemoglobin levels, no significant association could be established in both transcript groups. Data published previously in different studies regarding association between hematological variables at diagnosis and transcripts type remain controversial [38,39].

It is also believed that the type of transcript alone is not responsible for differences between the clinical and hematological parameters, and there must be some other factors that modify these parameters [34]. European Leukemia Net recommendations do not give any caution to patients with different transcripts and suggest that these variant translocations do not affect the prognosis of CML patients [16].

## 5 Conclusion

In this study group, 100% (70) of the CML patients were found to have typical BCR–ABL transcripts with preponderance of e14a2 subtype. The group of patients with e13a2 transcript presented with significantly higher mean leukocyte count compared to e14a2 group. There was gender skewed distribution with male prevalence in e14a2 and female predominance in e13a2 transcript. Difference in leukocyte counts at diagnosis and gender association in two groups might suggest a discrete pathobiology and phenotype of disease. Identification of transcript type is not only beneficial to monitor disease carefully in patients but can also help to select additional treatment regimen with specific transcript. Further studies with large sample size are needed to determine possible contribution of BCR–ABL transcripts types and the presentation of disease, which might help in prognosis and disease management.

## References

[1] Maru JE, Branford S. Current developments in molecular monitoring in chronic myeloid leukemia. Ther Adv Hematol. 2016;7:237–51. 10.1177/2040620716657994. PMC5026293 27695615

[2] Chasseriau J, Rivet J, Bilan F, Chomel JC, Guilhot F, Bourmeyster N, et al. Characterization of the different BCR–ABL transcripts with a single multiplex RT-PCR. J Mol Diagn. 2004;6:343–7. 10.1016/S1525-1578(10)60530-2 PMC1867492 15507673

[3] Kurzrock R, Kantarjian HM, Shtalrid M, Gutterman JU, Moshe T. Philadelphia chromosome-negative chronic myelogenous leukemia without breakpoint cluster region rearrangement: a chronic myeloid leukemia with a distinct clinical course. Blood. 1990;75:445–52. 2403827

[4] O’dwyer M. Multifaceted approach to the treatment of BCR–ABL-positive leukemias. Oncologist. 2002;7:30–8. 10.1634/theoncologist.7-suppl_1-30 11961207

[5] Kim JE, Yoon S, Choi BR, Kim KP, Cho YH, Jung W, et al. Cleavage of BCR–ABL transcripts at the T315I point mutation by DNAzyme promotes apoptotic cell death in Imatinib-resistant BCR–ABL leukemic cells. Leukemia. 2013;27:1650–8. 10.1038/leu.2013.60. 23434731

[6] Reena RMZ, Julia Munchar MJ, Salwati S, Zubaidah Z, Hamidah NH, Sharifah NA, et al. Detection of BCR/ABL gene in chronic myloid leukaemia: comparison of fluorescence in situ hybridisation (FISH), conventional cytogenetics and polymerase chain reaction (PCR) Techniques. Med Health. 2006;1:5–13.

[7] Sugimoto T, Ijima K, Hisatomi H, Murayama T, Mizuno I, Hato A, et al. Second case of CML with aberrant BCR–ABL fusion transcript (e8/a2) with insertion of an inverted ABL intron 1b sequence. Am J Hematol. 2004;77:164–6. 10.1002/ajh.20138. 15389825

[8] Deininger MWN, Goldman JM, Melo JV. The molecular biology of chronic myeloid leukemia. Blood. 2000;96:3343–56. 11071626

[9] Iqbal Z, Manzoor F, Iqbal M, Ali S, Sheikh N, Khan M, et al. Frequency of BCR–ABL fusion oncogene splice variants associated with chronic myeloid leukemia (CML). J Cancer Ther. 2011;2:176–80. 10.4236/jct.2011.22022.

[10] Burmeister T, Reinhardt R. A multiplex PCR for improved detection of typical and atypical BCR–ABL fusion transcripts. Leukemia Res. 2008;32:579–85. 10.1016/j.leukres.2007.08.017. 17928051

[11] Anand MS, Varma N, Varma S, Rana KS, Malhotra P. Cytogenetic and molecular analyses in adult chronic myelogenous leukaemia patients in north India. Indian J Med Res. 2012;135:42–8. 10.4103/0971-5916.93423 PMC3307183 22382182

[12] Laurent E, Talpaz M, Kantarjian H, Kurzrock R. The BCR Gene and Philadelphia Chromosome-positive Leukemogenesis. Cancer Res. 2001;61:2343–55. 11289094

[13] Balatzenko G, Vundinti BR, Margarita G. Correlation between the type of BCR–ABL transcripts and blood cell counts in chronic myeloid leukemia – a possible influence of mdr1 gene expression. Hematol Rep. 2011;3:5–9. 10.4081/hr.2011.e3. PMC3238477 22184525

[14] Bennour A, Saad A, Sennana H. Chronic myeloid leukemia: relevance of cytogenetic and molecular assays. Crit Rev Oncol/Hematol. 2016;97:263–74. 10.1016/j.critrevonc.2015.08.020. 26412717

[15] Chen Y, Wang H, Kantarjian H, Cortes J. Trends in chronic myeloid leukemia survival in the United States from 1975–2009. Leuk Lymphoma. 2013;54:1411–7. 10.1007/s13238-010-0016-z. PMC5525971 23121646

[16] Marzocchi G, Castagnetti F, Luatti S, Baldazzi C, Stacchini M, Gugliotta G, et al. Variant Philadelphia translocations: molecular-cytogenetic characterization and prognostic influence on front line imatinib therapy, a GIMEMA working party on CML analysis. Blood. 2011;117:6793–800. 10.1182/blood-2011-01-328294. 21447834

[17] Smith A, Roman E, Howell D, Jones R, Patmore R, Jack A. The haematological malignancy research network (HMRN): a new information strategy for population based epidemiology and health service research. Br J Haematol. 2009;148:739–53. 10.1111/j.1365-2141.2009.08010. PMC3066245 19958356

[18] Ashariati A, Ugroseno S. Profile of BCR–ABL transcript levels based on sokal prognostic score in chronic myeloid leukemia patients treated with imatinib. Acta Med Indones-Indones J Intern. 2013;45:108–13. 23770790

[19] Kuan JW, Michael MS. The epidemiology of chronic myeloid leukaemia in southern Sarawak, Borneo Island. Med J Malays. 2018;73:78–85. 29703870

[20] Tashfeen S, Ahmed S, Bhatti FA, Ali N. Real time polymerase chain reaction in diagnosis of chronic myeloid leukemia. J Coll Phys Surg Pak. 2014;24:190–3. 24613116

[21] Nguyen LT, Guo M, Naugler C, Rashid-Kolvear F. Incidence of chronic myeloid leukemia in Calgary, Alberta, Canada. BMC Res Notes. 2018;11:780. 10.1186/s13104-018-3890-8. PMC6211485 30382890

[22] Bhatti FA, Ahmed S, Ali N. Clinical and hematological features of 335 patients of chronic myelogenous leukemia diagnosed at single centre in Northern Pakistan. Clin Med Insights Blood Disord. 2012;5:16–24. 10.4137/CMBD.S10578.

[23] Khaled SAA, Nashwa MA, Aziz AE. Demographic, clinical, and hematologic characteristics of patients with chronic myeloid leukemia in Upper Egypt: association with treatment responses. Egypt J Haematol. 2015;40:195–200. 10.4103/1110-1067.170221.

[24] Deb P, Chakrabarti P, Chakrabarti S, Aich R, Nath U, Rav S, et al. Incidence of BCR–ABL transcript variants in patients with chronic myeloid leukemia: their correlation with presenting features, risk scores and response to treatment with imatinib mesylate. Indian J Med Paediatr Oncol. 2014;35:26–30. 10.4103/0971-5851.133707. PMC4080658 25006280

[25] Javed A, Mukhtar H, Kubra K, Lodhi S, Abaidullah S. Detection of BCR–ABL fusion gene and its transcript variants in chronic myeloid leukaemia patients – a multi-comparison study. JPMA. 2020;70:1748. 10.5455/JPMA.30606. 33159746

[26] Vasconcelos AP, Azevedo IF, Melo FCBC, Neves WB, Azevedo ACAC, Melo RAM. BCR–ABL1 transcript types showed distinct Laboratory characteristics in patients with chronic myeloid leukemia. Genet Mol Res. 2017;16(2):541. 10.4238/gmr16029541. 28437552

[27] Hassan R, Ramli M, Abdullah WZ, Mustaffa R, Ghazali S, Ankathil R, et al. One-step multiplex RT-PCR for detection of BCR/ABL gene in Malay patients with chronic myeloid leukaemia. AsPac J Mol Biol Biotechnol. 2008;16:41–4.

[28] Goh HG, Hwang JY, Kim SH, Lee YH, Kim YL, Kim DW. Comprehensive analysis of BCR–ABL transcript types in Korean CML patients using a newly developed multiplex RT-PCR. Transl Res. 2006;148:249–56. 10.1016/j.trsl.2006.07.002. 17145570

[29] Yaghmaie M, Ghaffari SH, Ghavamzadeh A, Alimoghaddam K, Jahani M, Mousavi SA, et al. Frequency of BCR–ABL fusion transcripts in iranian patients with chronic myeloid leukemia. Arch Iran Med. 2008;11:247–51. 18426313

[30] Mir R, Ahmad I, Javid J, Zuberi M, Yadav P, Shazia R, et al. Simple multiplex RT-PCR for identifying common fusion BCR–ABL transcript types and evaluation of molecular response of the a2b2 and a2b3 transcripts to Imatinib resistance in North Indian chronic myeloid leukemia patients. Indian J Cancer. 2015;52:314–8. 10.4103/0019-509X.176741. 26905124

[31] Sastre DA, Argarana CE, Heller VB, Gallo M, Fernández EN, Rodríguez CM. An analysis of multiplex-PCR in the detection of BCR–ABL transcripts in hematological disorders. Genet Mol Biol. 2007;30:520–3.

[32] Farhat-Maghribi S, Habbal W, Monem F. Frequency of BCR–ABL transcript types in Syrian CML patients. J Oncol. 2016;1–5. 10.1155/2016/8420853. PMC4904091 27313614

[33] Irshad S, Butt MA, Joyia A. Frequency of different BCR–ABL fusion transcripts in chronic myelogenous leukemia patients in Pakistan. IJAVMS. 2012;6:418–23. 10.5455/ijavms.25-1344841051.

[34] Filho TPA, Filho PAM, Barbosa MC, Dutra LLA, Castro MF, Duarte FBA, et al. Does BCR–ABL transcript type influence the prognosis of patients in chronic myelogenous leukemia chronic phase? Hematol Transfus Cell Ther. 2019;41:114–8. 10.1016/j.htct.2018.10.003. PMC6517615 31079657

[35] Achkar WA, Moassass F, Youssef N, Wafa A. Correlation of p210 BCR–ABL transcript variants with clinical parameters and disease outcome in 45 chronic myeloid leukemia patients. JBUON. 2016;21:444–9. 27273956

[36] Khazaal MS, Hamdan FB, Al‐Mayah QS. Association of BCR/ABL transcript variants with different blood parameters and demographic features in Iraqi chronic myeloid leukemia patients. Mol Genet Genomic Med. 2019;7:e809. 10.1002/mgg3.809. PMC6687619 31206255

[37] Jain P, Kantarjian H, Patel KP, Gonzalez GN, Luthra R, Shamanna RK, et al. Impact of BCR–ABL transcript type on outcome in patients with chronic phase CML treated with tyrosine kinase inhibitors. Blood. 2016;127:1269–75. 10.1182/blood-2015-10-674242. PMC4786836 26729897

[38] Polampalli S, Choughule A, Negi N, Shinde S, Baisane C, Amre P, et al. Analysis and comparison of clinicohematological parameters and molecular and cytogenetic response of two BCR/Abl fusion transcripts. Genet Mol Res. 2008;7(4):1138–49. 10.4238/vol7-4gmr485 19048492

[39] Mondal BC, Bandyopadhyay A, Majumdar S, Mukhopadhyay A, Chandra S, Chaudhuri U, et al. Molecular profiling of chronic myeloid leukemia in Eastern India. Am J Hematol. 2006;81:845–9. 10.1002/ajh. 16888785

